# GMS publishes your research findings – and makes the related research data available through Dryad

**DOI:** 10.3205/zma000976

**Published:** 2015-08-17

**Authors:** Ursula Arning

**Affiliations:** 1 ZB MED – Leibniz Information Centre for Life Sciences, Department Digital Publishing/GMS, Cologne, Germany

## Editorial

Since 2013, ZB MED – Leibniz Information Centre for Life Sciences has been actively committed to archiving and publishing research data. To offer authors an opportunity to publish research data in parallel with GMS publications, ZB MED has become a member of Dryad, an open access repository for the research data underlying articles in peer-reviewed journals. Presently, ten of the journals published by GMS have shown interest in making data accessible, including *GMS Zeitschrift für Medizinische Ausbildung* (ZMA).

**What is research data in medicine?** Research data in the field of medicine can be statistical data (e.g. from anonymized diagnostic findings), materials (e.g. International Statistical Classification of Diseases and Related Health Problems (ICD)), classifications and codes for diseases, pictures from imaging procedures (e.g. MRT), sensor data from biosignals or vital parameter measurements (e.g. ECG, EEG), biomaterial data from laboratory testing (e.g. blood samples, genome data), administrative patient information (e.g. from hospital information systems), as well as audio-visual data, models, and visualizations.

**Why should research data be available?** Repeated criticism is voiced in the sciences that research findings cannot be verified because the underlying research data have not been published. To aid transparency, good scientific practice should include making data publicly accessible. In addition, a duplication of work can be avoided if certain data do not need to be collected multiple times and can be re-used, even in new contexts. Another advantage is that data can be cited, potentially strengthening the author’s own scientific reputation. Plus, publishing the data can often fulfill the requirements or recommendations of different sponsors, such as DFG, BMBF, EU, NIH, or even the guidelines of the author’s own institution.

**Figure 1:**
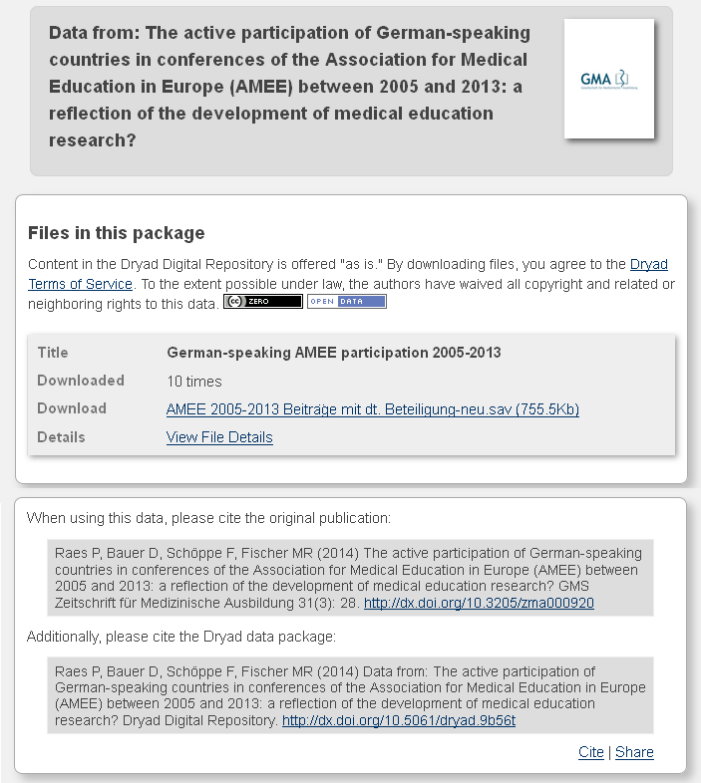
Cross-referencing example

**What are the advantages of making data accessible through Dryad?** With scientific societies and publishers among its members, Dryad is an internationally established repository. Its long-term preservation system, CLOCKSS, ensures permanent availability of the data. Dryad makes the data accessible under the terms of a Creative Commons Zero (CCO) license to allow for the widest possible use. In addition, the repository is widely disseminated making the research data easily discoverable. Through cross-referencing between a journal article and the related data set, the documents get new accessibility (see Figure 1 (Fig. 1)).

**Figure 2:**
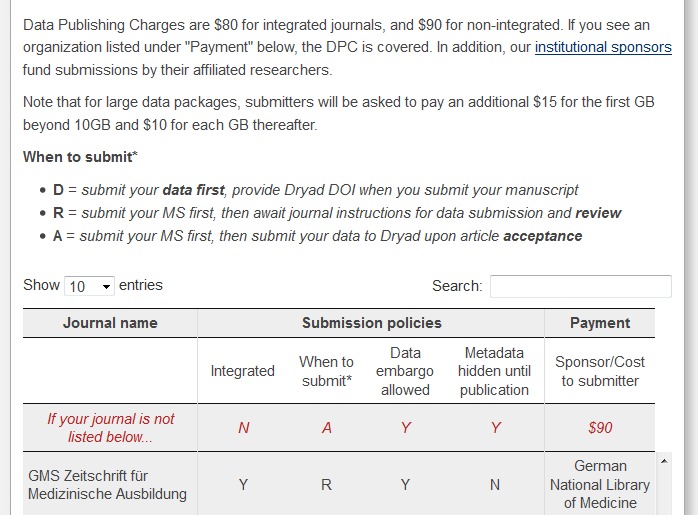
Information on submitting research data for ZMA (http://datadryad.org/pages/journalLookup)

Each data set receives its own permanent digital object identifier (DOI) allowing the data to be cited. Dryad also generates user statistics with which it is possible to ascertain the interest in the data and their potential scientific relevance (see Figure 2 (Fig. 2)).

**How does publication take place via GMS?** ZMA authors can indicate their wish to publish the underlying research data when submitting their manuscripts. If an author agrees, a message is sent directly to Dryad from the manuscript operating system, and a preliminary entry showing the manuscript’s metadata is created. The authors then receive an email with a direct link to this entry and can save data in any format and of any size. The data can thus be included in the review process via secured access.

Generally it must be noted that currently Dryad only accepts data in English, meaning German data will not be published. When submitting the research data, the metadata which are taken from the manuscript submission at GMS should be expanded to include other important details, such as co-authors, keywords, etc.

After nearly two years of Dryad membership, a clear discrepancy can be seen between a high percentage of affirmative declarations and very few actually published data sets. A rejection to publish research data has absolutely no influence on the review process.

As part of the submission process via the GMS manuscript operating system, we normally ask for the reasons why data upload to the Dryad repository was rejected, so that we can better focus our services on author needs. With the exception of “no data available”, the most common reasons at present have been a “mistrust” of the consequences of publishing and “authorship”, meaning doubts exist regarding data copyrights. In response to these concerns and other lack of information, ZB MED has put together publishing advice focusing on the topics of open access and open data. One basic component of this advising service are the FAQs listed on the ZB MED website since the beginning of 2015 (http://www.zbmed.de/en/publishing/publishing-advice-service/faqs/). In addition, ZB MED also offers consulting services and sessions on the topic of research data management (http://www.zbmed.de/en/publishing/publishing/research-data-management/).

**Figure 3:**
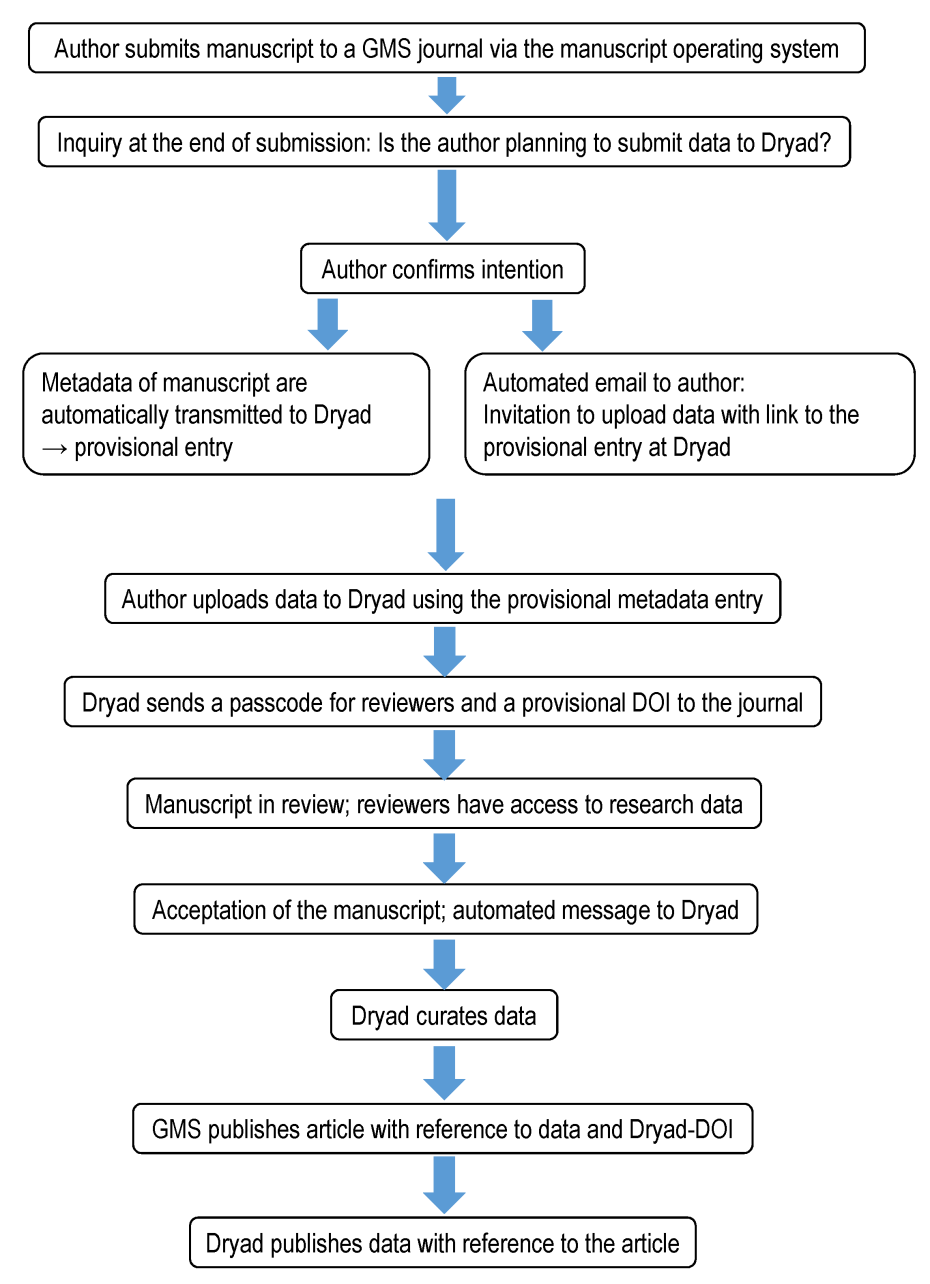
Dryad submission integration – workflow

Reasons such as “do not know how to upload data/Dryad” or “too difficult” are other important indicators for us on how our information needs to be constantly improved. This has prompted the GMS editorial office to write an explanatory text on the Dryad question that is asked when submitting manuscripts in order to make the process clearer. A tutorial is also being created to outline the process of publishing research data with Dryad when publishing an article with GMS. Ideally, the typical procedure for parallel publication of research data is presented in Figure 3 (Fig. 3).

In the future, ZB MED will continue to support open data and has committed itself to bear the costs until the end of 2016 (USD 80.00 per data set at Dryad). We would be very pleased to see more ZMA authors using this opportunity to publish research data in parallel with Dryad. 

## Competing interests

The author declares that she has no competing interests.

